# Editorial

**Published:** 1900-09-15

**Authors:** 


### EDITORIAL.

We are in receipt of a request, from one of our prominent dental journals, that we announce editorially the fact that one of its editors has gone to Paris for the special purpose of making a report of the proceedings of the International Dental Congress, which is to be published in due time in their journal. In a recent issue of the same journal the publishers print, in the advertising pages, a two page catechetical exposition of the rare personal equipment of its special correspondent for the expedition, with the usual invitation to subscribe.

Now, we are aware of the high professional and literary qualifications of the gentleman in question, and we have not the least doubt but that he will execute his mission to the entire satisfaction of his publishers and to the edification of the profession. And we do not want to appear mean or selfish by not responding to this courteous request, although it is asking for that which it is customary for publishers to receive a pecuniary consideration, neither are we envious of the ability of other journals to provide for their readers special reports of such meetings. In fact, we have tried to divest our mind of every possible unworthy motive for making any adverse comment. But we have been solicited to become a party to a proceeding which, because of the manner of its conduct, we are compelled to regard as menacing the highest ethical standards of our profession, if not an actual breach of its etiquette; hence, we feel not only justified but compelled to record a protest in behalf of dignified journalism. The entire procedure is so much like that of the professional advertiser or the secular newspaper that it is offensive, if not an actual breach of professional ethics, and can only be demoralizing in its tendency. We are quite sure the editor-in-chief of this journal did not sanction the methods used to announce this expedition, neither do we believe the special correspondent thus prominently advertised will relish the notoriety thus forced upon him in advance of the appearance in print of the results of his mission. We trust it is but an indiscreet display of excessive business enterprise on the part of the publishers; for we can not conceive that there is any business crisis threatening so valuable a journal as to justify its publishers in adopting business methods which are offensive to a profession which is obligated to maintain ethical standards equal to those of other professions with which it is classed. A profession's journals should not only promulgate that which is best in its literature, but they should also be conducted in strict accordance with its highest ethical sentiments. We trust our motive for entering this protest may not be misunderstood, at the same time we regret our incapacity for making an adequate pronouncement on the subject.

